# Portal venous velocity affects liver regeneration after right lobe living donor hepatectomy

**DOI:** 10.1371/journal.pone.0204163

**Published:** 2018-09-17

**Authors:** Chen-Tai Hou, Yao-Li Chen, Chia-Cheng Lin, Chen-Te Chou, Kuo-Hua Lin, Ping-Yi Lin, Ya-Lan Hsu, Chia-Bang Chen, Hui-Chuan Lin, Chih-Jan Ko, Su-Han Wang, Li-Chueh Weng, Chia-En Hsieh

**Affiliations:** 1 Surgical Critical Unit, Department of Surgery, Changhua Christian Hospital, Changhua, Taiwan; 2 Department of Surgery, Changhua Christian Hospital, Changhua, Taiwan; 3 School of Medicine, Kaohsiung Medical University, Kaohsiung, Taiwan; 4 Department of Radiology, Changhua Christian Hospital, Changhua, Taiwan; 5 Department of Biomedical Imaging and Radiological Science, National Yang-Ming Medical University, Taiper, Taiwan; 6 Transplant Medicine & Surgery Research Centre, Changhua Christian Hospital, Changhua, Taiwan; 7 Department of Nursing, Changhua Christian Hospital, Changhua, Taiwan; 8 Department of Nursing, Hung Kuang University, Taichung, Taiwan; 9 Department of Nursing, Graduate Institute of Clinical Medical Science, Chang Gung University, Taoyuan, Taiwan; 10 Graduate Institute of Clinical Medical Science, Chang Gung University, Taoyuan, Taiwan; Texas A&M University, UNITED STATES

## Abstract

**Objectives:**

We investigated whether chronological changes in portal flow and clinical factors play a role in the liver regeneration (LR) process after right donor-hepatectomy.

**Materials and methods:**

Participants in this prospective study comprised 58 donors who underwent right donor-hepatectomy during the period February 2014 to February 2015 at a single medical institution. LR was estimated using two equations: remnant left liver (RLL) growth (%) and liver volumetric recovery (LVR) (%). Donors were classified into an excellent regeneration (ER) group or a moderate regeneration (MR) group based on how their LR on postoperative day 7 compared to the median value.

**Results:**

Multivariate analysis revealed that low residual liver volume (OR = .569, 95% CI: .367– .882) and high portal venous velocity in the immediate postoperative period (OR = 1.220, 95% CI: 1.001–1.488) were significant predictors of LR using the RLL growth equation; high portal venous velocity in the immediate postoperative period (OR = 1.325, 95% CI: 1.081–1.622) was a significant predictor of LR using the LVR equation. Based on the two equations, long-term LR was significantly greater in the ER group than in the MR group (p < .001).

**Conclusion:**

Portal venous velocity in the immediate postoperative period was an important factor in LR. The critical time for short-term LR is postoperative day 7; it is associated with long-term LR in donor-hepatectomy.

## Introduction

Many adult-to-adult living donor liver transplantation centers perform left lobe grafts because they are deemed to be safer than other methods. However, an increasing number of studies have shown that up to 70% of the liver volume can be removed from the donor without compromising patient safety [1–3]. In our institution, we believe that if the liver quality and the regeneration rate are good then donor safety can be ensured by preserving approximately one-third of the total liver volume.

After donor hepatectomy, the residual liver undergoes the process of regeneration. The mechanisms governing the process are not completely understood but are known to involve various cytokines, growth factors, and transcription factors [4–7]. Some authors have proposed that the regeneration process is triggered by postoperative hemodynamic changes [4, 6]. In other studies, it has been reported that the most significant increase in liver volume occurs during postoperative day 7 [8–12]. However, the role hepatic blood flow plays in liver regeneration remains unclear [4]. In this study, we investigated whether chronological changes in portal flow and clinical factors play a role in the liver regeneration process after right donor hepatectomy.

## Materials and methods

### Donors

The eligibility criteria for subjects to undergo right hepatectomy included the preoperative evaluations of psychological condition, social assessment, and general physical condition. We considered an estimated remnant liver volume >30–35% to be optimal in the donors, and the graft-recipient weight ratio (GRWR) was 0.8% to 1% in the recipients [2, 3]. Adult-to-adult liver transplantation was most preservation of the middle hepatic vein undergo right hepatectomy. The study protocol and the surgical procedure were approved by the Institutional Review Board of Changhua Christian Hospital (No. 140708). None of the transplant donors were from a vulnerable population and all donors provided written informed consent that was freely given.

### Inclusion and exclusion criteria

During the period February 2014 to February 2015, a total of 59 living donors underwent hepatectomy with preservation of the middle hepatic vein at Changhua Christian Hospital. Donors with a left hepatectomy were excluded. A total of 58 donors fulfilled the inclusion criteria.

### Measurement of liver & spleen volume

Computed tomography scans were analyzed using 3D reconstruction software to estimate liver and spleen volumes (IQQA-Liver, EDDA Technology, Inc.). Residual liver volume ratio was defined as the ratio of remnant left liver volume and preoperative total liver volume (RLL/TLV). The rate of spleen growth on postoperative day 7 (%) was defined as (spleen volume at POD 7- preoperative spleen volume) /preoperative spleen volume x 100. We measured liver regeneration volume in postoperative week 1, postoperative month 1, postoperative month 3, and postoperative year 1.

### Regeneration of the liver was estimated using two equations

Remnant left liver (RLL) growth rate (%) was defined as (liver volume at POD X- preoperative RLL) /preoperative RLL x 100.

Liver volumetric recovery (LVR) rate (%) of remnant liver was defined as (current liver volume at POD X/ preoperative total liver volume) x100.

### Doppler measurements

All Doppler studies were performed with color and pulsed Doppler units using an S4-2 2 MHz sector-phased array transducer coupled to a Philips HD11 XE Ultrasound System (Philips Medical Systems B.V., Netherlands). We measured mean portal venous velocity (cm/sec) in the left branch of the portal vein before the operation, immediately after the operation, and on POD 1, 3, 5, and 7. Mean portal venous velocity was measured by tracing representative waves and was corrected by the angle (30° to 60°) between the long axis of the left portal vein.

### Design

Based on postoperative day 7 measurements, patients with liver regeneration (RLL growth and LVR) greater than or equal to the median value were included in the excellent regeneration (RLL-ER or LVR-ER) group and those with a regeneration rate less than the median value were included in the moderate regeneration (RLL-MR or LVR-MR) group.

The presence of regeneration was correlated with portal venous velocity or clinical factors, including age, gender, hepatic steatosis, body mass index (BMI), residual liver volume, spleen volume, and platelet count. Outcomes recorded included aspartate aminotransferase (AST), alanine transaminase (ALT), total bilirubin, ascites amount during hospital stay, complication with bile leakage, and length of stay.

### Fluid management and nutritional support after hepatectomy

A central venous catheter was inserted into the internal jugular vein in the operative room. The purpose of establishing a central venous catheter was to facilitate fluid management and monitor hemodynamic status, which was kept at 6-10cmH_2_O. The line was removed on POD 4. All donors received peripheral parenteral nutrition for four consecutive days after the operation. Peripheral parenteral nutrition supply in right lobe donors has been associated with greater recovery of liver function and a decreased rate of complications [13]. Donors were allowed to eat by postoperative day 3–5 when bowel sounds and flatus were noted.

### Statistical analysis

Results were collected for analysis. Continuous variables are presented as mean ± standard deviation (SD). The Pearson chi-square test, the Fisher’s exact test, independent t-tests, the Mann-Whitney test and repeated measures ANOVA were used to examine differences in demographic and clinical characteristics between the excellent regeneration group and the moderate regeneration group. Significant variables in the univariate analyses were evaluated with multivariate logistic regression to identify the most important factors. P values <0.05 were considered to indicate statistical significance. All statistical analyses were performed using IBM SPSS, version 20.0.

## Results

Mean donor RLL growth was 74.20±28.70% (range, 0.42% to 138.20%) and median donor RLL growth was 72.5%. Table 1 shows the demographic data and clinical features of all donors. Patients were stratified into one of two groups depending on their RLL growth relative to the median, i.e., a moderate regeneration group (RLL growth <72.5%, n = 29; RLL-MR) and an excellent regeneration group (RLL growth ≥ 72.5%, n = 29; RLL-ER). We found that patients in the RLL-ER group had a significantly lower mean residual liver volume (% or cm^3^) (p< .001) than patients in the RLL-MR group. Table 2 shows the post-hepatectomy demographic data and clinical features. We found that patients in the RLL-ER group had a significantly higher mean RLL growth on POD 7 (p< .001) and significantly higher spleen growth on postoperative day 7 (p = .020) than patients in the RLL-MR group, and that the RLL-ER group had a significantly lower mean postoperative platelet count (p = .017) than patients in the RLL-MR group.

**Table 1:** Comparisons of preoperative demographic data and clinical features of two groups of liver donors.

Demographic and clinical features	Remnant left liver growth equation		*p*	Liver volumetric recovery equation		*p*	Total
	MR (n = 29)	ER (n = 29)		MR (n = 28)	ER (n = 30)		N = 58
	Mean±SD (range)	Mean±SD (range)		Mean±SD (range)	Mean±SD (range)		Mean±SD (range)
Age (years)	31.07±9.48 (19–61)	31.28±8.13 (20–49)	.929	29.71±7.25 (19–48)	32.53±9.88 (20–61)	.268	31.17±8.75 (19–61)
Body Mass Index	23.63±3.56 (18.97–30.82)	23.91±3.51 (18.38–34.48)	.765	24.21±3.65 (19.07–30.82)	23.36±3.38 (18.38–34.48)	.315	23.77±3.51 (18.38–34.48)
RLV (%)	39.54±4.22 (32.23–49.43)	35.34±3.05 (30.00–41.22)	< .001	37.47±4.56 (30.00–49.43)	37.41±3.96 (30.98–46.45)	.950	37.44±4.22 (30.00–49.43)
RLV (cm^3^)	555.89±115.21 (364.2–888.3)	433.77±102.48 (236.6–655.1)	< .001	532.95±131.65 (364.2–888.3)	459.23±107.49 (236.6–655.1)	.023	191.83±124.39 (236.6–888.3)
TLV (cm^3^)	1362.02±233.63 (1067.4–1933.6)	1249.53±273.70 (716.0–1765.0)	.098	1440.82±257.09 (1067.4–1933.6)	1270.10±230.11 (716.0–1765.0)	.006	1305.78±258.51 (716.0–1933.6)
Spleen volume (cm^3^)	173.21±67.60 (56.0–370.0)	179.24±61.10 (95.0–308.0)	.723	167.57±59.44 (56.0–370.0)	184.30±67.86 (95.0–308.0)	.455	176.22±63.93 (56–370)
Preoperative PLT (x 1000 / μL)	258.10±53.03 (182.0–408.0)	234.31±41.01 (145.0–300.0)	.061	265.57±48.36 (182.0–408.0)	228.13±41.76 (145.0–300.0)	.003	246.21±48.49 (145–408)
	(%)	(%)		(%)	(%)		
Gender			.430			.118	
Male	14(48.3)	17(58.6)		12(42.9)	19(63.3)		27(46.6)
Female	15(51.7)	12(41.4)		16(57.1)	11(36.7)		31(53.4)
Hepatic steatosis			.738			.835	
>30%	6(20.7)	5(17.2)		5(17.9)	6(20.0)		11(19.0)
<30%	23(79.3)	24(82.8)		23(82.1)	24(80.0)		47(81.0)

Note. MR, moderate regeneration; ER, excellent regeneration; RLV, residual liver volume; TLV, total liver volume; PLT, platelet.

**Table 2:** Comparisons of postoperative demographic data and clinical features of two groups of liver donors.

Demographic and clinical features	Remnant left liver growth equation		*p*	Liver volumetric recovery equation		*p*	Total
	MR (n = 29)	ER (n = 29)		MR (n = 28)	ER (n = 30)		N = 58
	Mean±SD (range)	Mean±SD (range)		Mean±SD (range)	Mean±SD (range)		Mean±SD (range)
RLL growth (%)	50.30±16.44 (0.42–72.13)	98.10±14.93 (72.84–138.20)	< .001				74.20±28.70 (0.42–138.20)
LVR (%)				59.61±4.85 (46.07–66.14)	73.40±5.06 (67.21–85.49)	< .001	66.74±8.51 (46.07–85.49)
Blood loss (ml)	198.28±302.22 (50.00–1700.00)	229.31±205.95 (50.00–1000.00)	.649	216.07±340.22 (50.00–1700.00)	211.67±147.79 (50.00–600.00)	.115	23.77±3.51 (18.38–34.48)
Postoperative PLT (x 1000 / μL)	181.52±40.46 (182.0–408.0)	158.10±31.07 (100.0–212.0)	.017	184.29±36.95 (118–256)	156.30±33.53 (100–220)	.004	169.81±37.652 (100–256)
Spleen growth on POD 7 (%)	36.72±14.85 (9.89–68.14)	49.06±23.29 (13.10–89.40)	.020	41.23±20.88 (13.41–89.40)	44.44±20.04 (9.89–82.61)	.474	42.89±20.34 (9.89–89.40)
Ascites (ml)	1039.59±546.18 (169–2453)	1162.34±592.91 (260–2800)	.457	1028.61±563.10 (254–2335)	1168.50±673.90 (169–2800)	.367	246.21±48.49 (145–408)
Length of stay (day)	9.28±0.65 (9.0–12.0)	10.31±4.03 (8.0–30.0)	.182	9.21±0.42 (9.0–10.0)	10.33±3.98 (8.0–30.0)	.366	
	(%)	(%)		(%)	(%)		
Bile leakage			.300			.113	
No	28(96.6)	26(89.7)		28(100)	26(86.7)		27(46.6)
Yes	1(3.4)	3(10.3)		0(0)	4(13.3)		31(53.4)

Note. MR, moderate regeneration; ER, excellent regeneration; RLL, remnant left liver; LVR, liver volumetric recovery; PLT, platelet; POD, postoperative day.

Mean donor LVR was 66.7±8.5% (range, 46.1% to 85.5%) and median donor LVR was 67.2%. Patients were stratified into one of two groups depending on their LVR relative to the median, i.e., a moderate regeneration group (LVR <67.2%, n = 28; LVR-MR) and an excellent regeneration group (LVR ≥ 62.7%, n = 30; LVR-ER). Table 1 shows the demographic data and clinical features of all donors. We found that patients in the LVR-ER group had a significantly lower preoperative platelet count (p = .003), mean residual liver volume (p = .023) and total liver volume (p = .006) than patients in the LVR-MR group. Table 2 shows post-hepatectomy demographic data and clinical features. We found that patients in the LVR-ER group had a significantly higher mean LVR on POD 7 (p< .001) than patients in the LVR-MR group, and that the LVR-ER group’s patients had a significantly lower mean postoperative platelet count (p = .004) than patients in the LVR-MR group.

**Fig 1:**
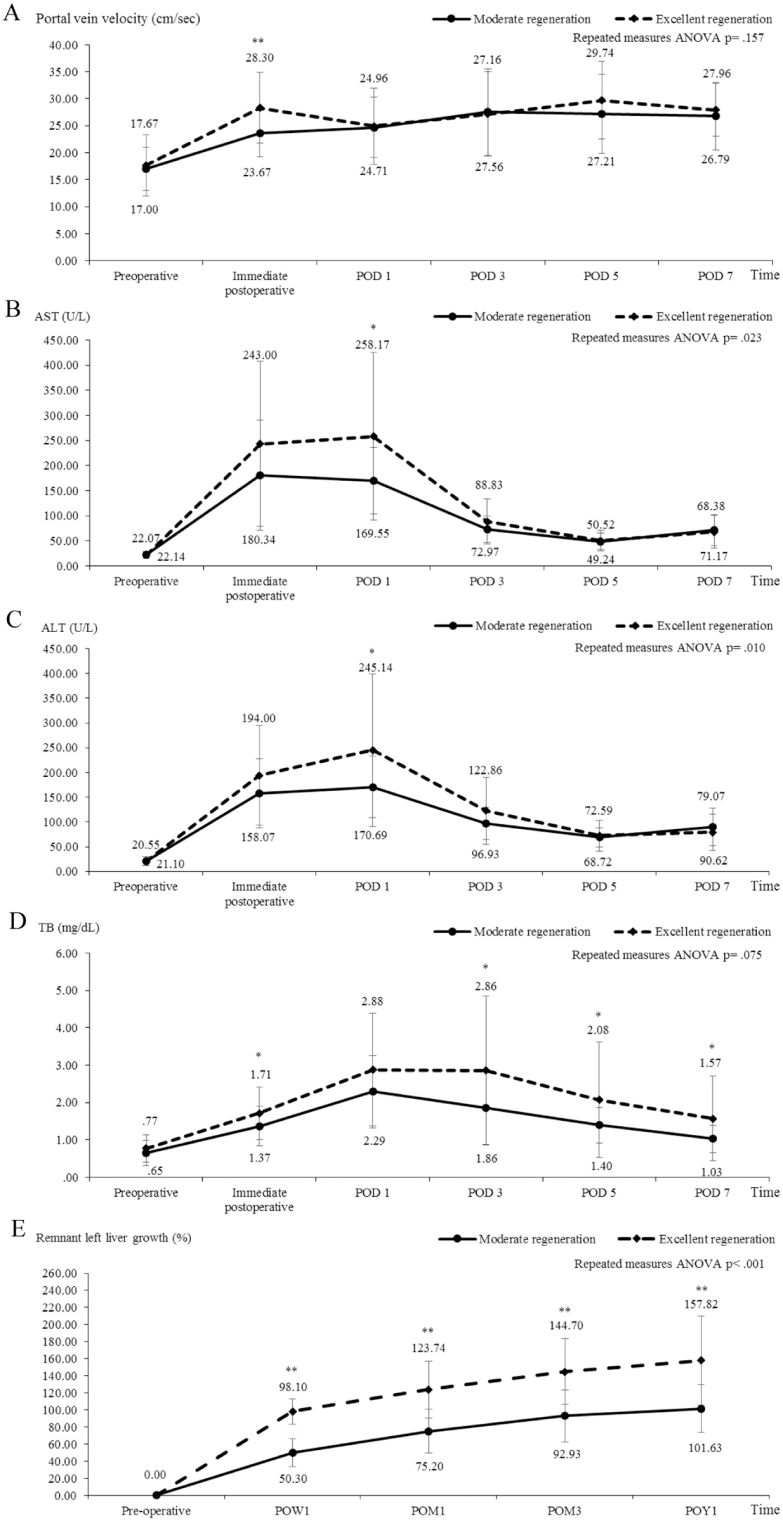
Comparisons of clinical parameters in right donor hepatectomy with remnant left liver growth equation. All donors’ mean aspartate aminotransferase (AST), alanine transaminase (ALT), total bilirubin (TB) level, portal venous velocity, and liver growth showed a trend of continuous by remnant left liver growth equation. All patients in the RLL-ER group had a significantly higher portal venous velocity immediately post-operation (A, p< .003). AST (B, p = .023), ALT (1C, p = .010) levels and RLL growth (D, p< .001) were significantly higher in the RLL-ER group than in the RLL-MR group by repeated measures ANOVA. (*p < .05, **p < .01). Note: AST, aspartate aminotransferase; ALT, alanine transaminase; TB, total bilirubin; POD, postoperative day; POW, postoperative week; POM, postoperative month; POY, postoperative year.

**Fig 2:**
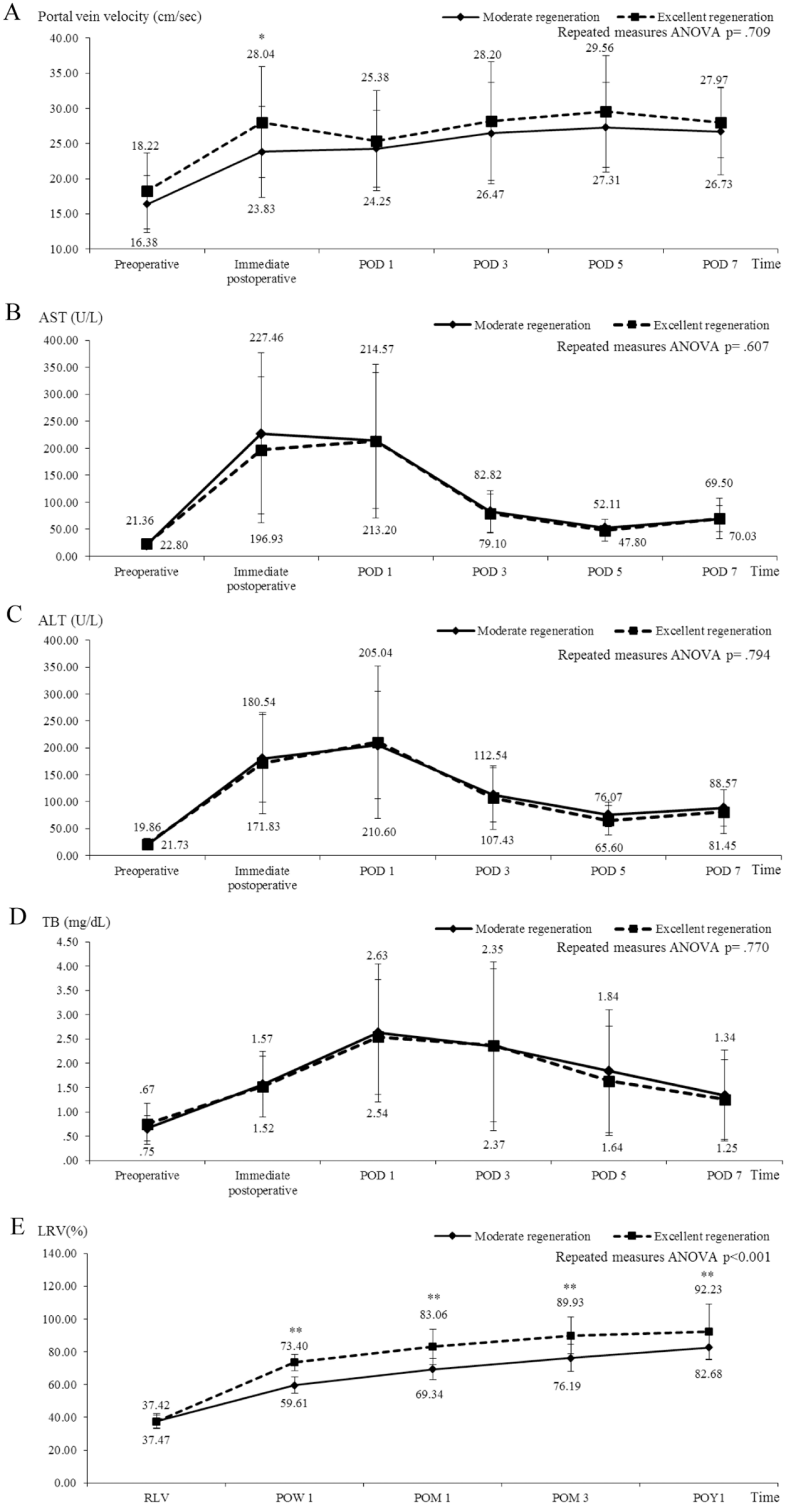
Comparisons of clinical parameters in right donor hepatectomy with liver volumetric recovery equation. Using the LVR equation, we found that patients in the LVR-ER group had a significantly higher portal venous velocity immediately post-operation (A, p = .039). AST, ALT, and TB levels showed no significant differences between the two groups. LVR (E, p< .001) levels were significantly higher in the LVR-ER group than in the LVR-MR group by repeated measures ANOVA. (*P < .05, **P < .01). Note: AST, aspartate aminotransferase; ALT, alanine transaminase; TB, total bilirubin; POD, postoperative day; POW, postoperative week; POM, postoperative month; POY, postoperative year.

All donors’ mean AST, ALT, total bilirubin level, portal venous velocity, and liver growth showed a trend of continuous by the RLL growth equation (Fig 1). We found by the Mann-Whitney test that patients in the RLL-ER group had significantly higher measurements than patients in the RLL-MR group with regard to portal venous velocity immediately post-operation (p< .003), AST on POD 1 (p = .012), ALT on POD 1 (p = .021), and total bilirubin immediately post-operation (p = .042), as well as on POD 3 (p = .019), POD 5 (p = .031), and POD 7 (p = .022). AST (p = .023) and ALT (p = .010) levels were significantly higher in the RLL-ER group than in the RLL-MR group by repeated measures ANOVA. A high liver enzyme level after hepatectomy was significantly correlated with lower residual liver volume (Mean: 35.34±3.05%). Using the LVR equation, we found that patients in the LVR-ER group had a significantly higher portal venous velocity immediately post-operation (p = .039). AST, ALT, and total bilirubin levels showed no significant differences between the two groups(Fig 2).

Based on the RLL growth equation, postoperative week 1 (p< .001), postoperative month 1 (p< .001), postoperative month 3 (p< .001), and postoperative year 1 (p< .001) showed significantly greater liver regeneration in the RLL-ER group than in the RLL-MR group by the Mann-Whitney test and by repeated measures ANOVA (p< .001) (Fig 1E). In the LVR equation, postoperative week 1 (p< .001), postoperative month 1 (p< .001), postoperative month 3 (p< .001), and postoperative year 1 (p = .008) showed significantly greater liver regeneration in the LVR-ER group than in the LVR-MR group by the Mann-Whitney test and by repeated measures ANOVA (p< .001) (Fig 2E). In the two equations, we found that long-term outcomes were significantly associated with better liver regeneration in the first week (p< .001).

Multivariate analysis revealed that low residual liver volume (OR = .495, 95% CI: .291– .843) and high portal venous velocity in the immediate postoperative period (OR = 1.635, 95% CI: 1.109–2.412) were significant predictors of RLL growth. High portal venous velocity in the immediate postoperative period (OR = 1.325, 95% CI: 1.083–1.622) was a significant predictor of LVR (Table 3).

**Table 3:** Logistic regression analysis of factors affecting remnant left liver growth and liver volumetric recovery.

Variable	Remnant left liver growth equation						Liver volumetric recovery equation					
	Univariate analysis			Multivariate analysis			Univariate analysis			Multivariate analysis		
	OR	95% CI	*p*	OR	95% CI	*p*	OR	95% CI	*p*	OR	95% CI	*p*
Gender (Male)	1.518	.538–4.284	.431	-	-	-	2.303	.802–6.610	.121		-	-
Hepatic steatosis	.799	.214–2.982	.738	-	-	-	1.150	.308–4.294	.835	-	-	-
Age	1.003	.945–1.064	.928	-	-	-	1.040	.976–1.107	.224	-	-	-
BMI	1.023	.882–1.187	.760	-	-	-	.931	.800–1.083	.354	-	-	-
RLV	.721	.595-.873	.001	.495	.291-.843	.010	.997	.882–1.127	.961	-	-	-
Spleen volume	1.002	.993–1.010	.717	-	-	-	1.004	.996–1.013	.320	-	-	-
Preoperative PLT	.989	.977–1.001	.070	-	-	-	.980	.966-.994	.007	-	-	-
Blood loss	1.000	.998–1.003	.647	-	-	-	1.000	.998–1.002	.948	-	-	-
Postoperative PLT	.982	.966-.997	.022	-	-	-	.977	.961-.994	.008	-	-	-
Spleen growth POD 7	1.033	1.004–1.064	.025	-	-	-	1.008	.982–1.034	.547	-	-	-
Ascites	1.000	.999–1.001	.450	-	-	-	1.000	1.000–1.001	.390	-	-	-
PVV (Immediate postoperative)	1.176	.044–1.324	.007	1.635	1.109–2.412	.013	1.168	1.039–1.313	.010	1.325	1.083–1.622	.006
Peak AST	1.007	1.001–1.013	.026	-	-	-	1.000	.996–1.004	.969	-	-	-
Peak ALT	1.006	1.001–1.012	.033	-	-	-	1.000	.996–1.005	.861	-	-	-
Peak TB	1.579	1.049–2.377	.029	-	-	-	.951	.636–1.423	.807	-	-	-

Note. BMI, body mass index; RLV, residual liver volume; PLT, platelet; PVV, portal venous velocity; POD, postoperative day; AST, aspartate aminotransferase; ALT, alanine transaminase, TB, total bilirubin.

## Discussion

Evaluation of liver regeneration was performed using two equations, RLL growth (%) and LVR (%). RLL growth was calculated based on the rate of regenerated volume compared to the residual liver volume, but LVR was calculated based on the current volume compared to the preoperative total liver volume. The use of two equations to evaluate liver regeneration decreased any potential bias in the results. We chose postoperative day 7 to perform evaluations because liver regeneration has been shown to be fastest within the first week after living donor hepatectomy [8, 9, 11]. Patients with excellent regeneration (RLL growth or LVR) on postoperative day 7 tend to have better regeneration in the first postoperative year. The two equations were measuring tools that were used to identify excellent regeneration. However, excellent regeneration was more accurately predicted by RLL growth rate. Patients with liver regeneration (RLL growth or LVR) greater than or equal to the median value on postoperative day 7 were included in the excellent regeneration group, and those with liver regeneration less than the median value were included in the moderate regeneration group. We selected neither donors nor different surgical approaches. We found that when liver regeneration was better, small residual liver volume or total liver volume were maybe important factors. When living donor hepatectomy considered an estimated remnant liver volume >30–35%, liver regeneration could be safety outcome. Previous studies found a change in portal venous velocity after hepatic resection, and showed higher levels preoperatively than at any postoperative time point in all donors [14, 15]. In our study postoperative portal venous velocity at any time point was higher, up to a mean of 24 to 25 cm/sec, compared with the preoperative level in the left branch, with a mean portal venous velocity of 17.33±4.84 cm/sec. The overall mean portal venous velocity increased by up to 40–60% in our study. This implies that hemodynamics in the portal vein, i.e., portal venous velocity, play a role in liver regeneration. We found that portal venous velocity was higher in the excellent regeneration group than in the moderate regeneration group at all time points, especially immediately after operation, which was associated with a significantly higher portal venous velocity level. Park et al. found that the short-term graft regeneration ratio in recipients was positively correlated with portal flow velocity index and portal flow volume index at POD 1 [16]. Eguchi et al. reported that increased portal venous velocity on POD 7 was positively correlated with graft regeneration at 1 month in recipients [15]. According to the aforementioned studies, when portal venous velocity was higher after hepatectomy, there was a positive correlation with excellent regeneration. Portal venous velocity change after hepatectomy thus appears to be a crucial factor in liver recovery.

The effect of hemodynamic changes in the portal vein on the initiation and promotion of liver regeneration after hepatectomy has been extensively studied in human and animal models. Any factor that is capable of influencing hepatic vascular resistance can possibly modify portal blood flow. Cytokines play an important role by inducing hepatocytes to enter the cell cycle and to respond to the mitogenic effects of growth factors [5]. The main participants in the cytokine network are tumor necrosis factor (TNF) and interleukin-6 (IL-6). These cytokines induce genes that synthesize other peptides in the cytokine family and induce several mediators, such as prostanoids, leukotrienes, nitric oxide (NO), reactive oxygen species, and platelet-activating factor, all of which can affect vascular function. Cytokines have also been shown to induce the overexpression of TNF-α, IL-6, and IL-10, which lead to vasoconstriction in human internal thoracic arteries [17]. In arterioles, however, IL-1, IL-6, prostacyclin-2, and NO from inducible nitric oxide synthase have been shown to contribute to vasodilation [18, 19]. TNF-α causes vasodilation possibly through a mechanism involving cyclooxygenase and nitric oxide synthase [20]. We believe that the rapid increase in portal vein flow reflects a rapid hepatic vascular vasodilation. Peak serum levels of TNF-α and IL-6 were observed during the first 2–6 hours after partial hepatectomy in mice; the levels decreased gradually 6 hours after partial hepatectomy [21]. In our study the short-term regeneration ratio of donors was positively correlated with the portal flow velocity index immediately after the operation. Sasturkar et al. reported that the peak level of IL-6 and TNF-α were observed on POD 1, the roles of TNF-α and IL-6 were necessary for proper human liver regeneration after partial hepatectomy in healthy liver donors [22]. Chae et al. reported that higher serum IL-6 and TNF-α levels were associated with increased early graft regeneration after living donor liver transplantation [23]. IL-6 and TNF-α were important mediators in early regeneration.

Several animal studies have demonstrated that portal vein flow increases immediately after hepatectomy [24, 25], possibly due to factors such as the induction of urokinase plasminogen activator gene expression and the activation of hepatocyte growth factor [7]. Mueller et al. found that the induction of immediate-early genes, including early growth response gene-1, type-1 plasminogen activator inhibitor, and phosphatase of regenerating liver-1 is related to increased portal vein flow [26].

Although some of these mediators might be responsible for lowering hepatic vascular vasodilation in donors after hepatectomy, the exact mechanism governing the increase in portal vein blood flow after liver resection remains unclear. Iimuro et al. found that regeneration after hepatic resection was asymmetric in patients with diseased livers and that higher portal flow in remnant liver segments correlated with better regeneration [27]. Finally, one of the main strengths of this study was the large number of cases included in the analysis (58 cases), which allowed a meaningful investigation of the associations between portal venous velocity and liver regeneration. We believe that these findings may help living donor liver transplantation teams improve patient outcomes. This study has some limitations. First, there is the lack of biological parameters to evaluate possible mechanisms which link liver regeneration to clinical outcomes. In the present study, we did not measure the cytokine levels in serum of donors and preoperative liver fibrosis was not evaluated in donors. Future studies to evaluate such data are needed.

## Conclusion

Rapid liver regeneration in right lobe living donor hepatectomy occurs on postoperative day 7. High portal venous velocity in the immediate postoperative period was an important hemodynamic factor in the short-term regeneration process after right donor hepatectomy. The critical time for short-term regeneration is postoperative day 7; it is associated with future long-term regeneration in donor-hepatectomy. Unfortunately, a high liver enzyme level after hepatectomy was significantly correlated with lower RLV, which may be related to greater liver injury or dysfunction, and therefore it is imperative that liver function in donors be closely monitored.
